# Cochlear Implantation and Perioperative Management in Autoimmune Inner Ear Disease: A Systematic Review and Meta-Analysis

**DOI:** 10.1097/ONO.0000000000000006

**Published:** 2021-12-09

**Authors:** Nikita Deshpande, Nathan Aminpour, Hui Cheng, J. Dixon Johns, Michael Hoa

**Affiliations:** 1 Georgetown University School of Medicine, Washington, DC; 2 Bioinformatics and Biostatistics Collaboration Core, National Institute on Deafness and Other Communication Disorders, NIH, Bethesda, MD; 3 Department of Otolaryngology—Head and Neck Surgery, MedStar Georgetown University Hospital, Washington, DC.; 4 Auditory Development and Restoration Program, Otolaryngology Surgeon-Scientist Program, National Institute on Deafness and Other Communication Disorders, NIH, Bethesda, MD

**Keywords:** Autoimmune inner ear disease, Cochlear implant, intraoperative management, Pure-tone average, Speech perception

## Abstract

**Objective:**

To assess outcomes in autoimmune inner ear disease (AIED) after cochlear implantation (CI) through systematic review and meta-analysis.

**Databases Reviewed:**

PubMed, MedLine, Embase, and CINAHL

**Methods:**

Databases were queried for inclusion of AIED patients who underwent CI with outcomes recorded ≥3 months postoperatively. We examined demographics, pure-tone average (PTA), speech perception, preoperative imaging, intraoperative management, and postoperative complications.

**Results:**

Twenty-six articles encompassing 124 patients met inclusion criteria. Mean implantation age was 26.2 years (range 4–65 years) with average length of follow-up at 28.2 months (range 3–120 months). Meta-analysis demonstrated significant improvement in speech perception following CI. There was a statistically significant improvement in speech recognition score (SRS) (standard mean difference [SMD] = 6.5, 95% confidence interval [CI], 4.8-8.0, *P* < 0.0001) as well as word recognition score (WRS) (SMD = 5.5, 95% CI, 4.2-6.8, *P* < 0.0001) after CI. Anomalous preoperative radiologic manifestations were reported by 57.7% (15/26) studies. Disease activity–related intraoperative adjustment was noted in 57.7% (15/26) studies; common consequences were cochlear drill-out (53.3%), difficult round window insertion (26.7%), and scala vestibuli insertion (26.7%). Frequent postoperative complications noted in 26.9% (7/26) studies included systemic AIED flares (71.4%) and wound healing delay (42.9%).

**Conclusion:**

Findings of this systematic review of AIED cochlear implant literature demonstrate a lack of consistent reporting standards for PTA and speech perception as well as a lack of long-term follow-up. Despite these findings, meta-analysis suggests that CI is a viable treatment for improving speech perception in AIED patients.

## Abstract 2

Supplemental Digital Content is available in the text.

Autoimmune inner ear disease (AIED) is an otologic condition characterized by rapidly progressive bilateral sensorineural hearing loss (SNHL) with positive response to immunosuppressant therapy (1,2). Preliminary literature describing AIED was published in 1979 by McCabe et al (2); however, given low prevalence of AIED in the general population (1 in 5000–10,000), various aspects of this disorder such as disease pathogenesis remain poorly studied and understood (1,3). Current prevailing theory suggests that the overall disease entity of AIED can present itself as primary AIED in isolation, or secondary AIED within the context of systemic autoimmune disease such as systemic lupus erythematosus, rheumatoid arthritis, Cogan syndrome, Sweet’s syndrome, relapsing polychondritis, or eosinophilic granulomatosis with polyangiitis (4–12). Mainstay treatment for AIED is administration of corticosteroids, yet only about 50% of patients have shown positive corticosteroid therapy response in prior retrospective reviews and clinical trials (13–15). As a result, patients who develop moderate to severe SNHL are considered candidates for cochlear implantation (CI) (12).

Over the last 2 decades, several case reports and retrospective reviews have reported on post-CI outcomes for AIED patients (4–12,16–30). Despite growing literature on CI in patients with AIED, there are discrepancies in the reporting of postimplantation audiologic data and perioperative outcomes. To the best of our knowledge, this is the first study to systematically review and synthesize post-CI audiologic outcomes and perioperative complications for patients with AIED. Our study analyzes existing literature on CI outcomes in AIED patients to better understand the audiometric, speech perception, and perioperative management outcomes.

## MATERIALS AND METHODS

### Systematic Review

**FIG. 1.**
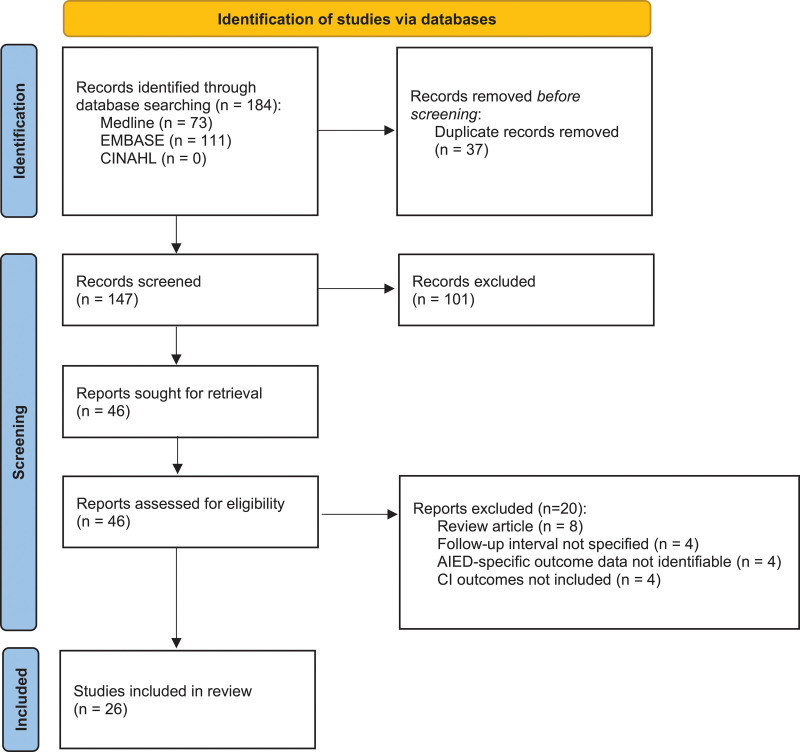
PRISMA flow diagram showing manuscript screening and selection criteria. AIED indicates autoimmune inner ear disease; CI: cochlear implantation.

A systematic review was conducted using PubMed, Medline, and Embase libraries on March 27, 2021 and CINAHL on October 5, 2021, to isolate primary literature on patients diagnosed with AIED who subsequently underwent CI (Fig. 1). See Supplemental Appendix 1, http://links.lww.com/ONO/A0, for full database search strategy. All 4 databases were queried in consultation with a medical librarian using the following keywords: *autoimmune inner ear disease, immune-mediated inner ear disease, autoinflammatory disorder, Cogan’s syndrome, Sweet’s syndrome, relapsing polychondritis, inflammatory bowel disease, cochlear implantation, correction of hearing impairment,* and *cochlear prosthesis*. All reviews, commentaries, editorials, unpublished studies, and abstracts were excluded. Our study protocol was registered in the PROSPERO prospective database of systematic reviews (CRD42021259767).

The PICOTS statement for this study is as follows: participants: patients with AIED; intervention: CI; comparisons: preoperatively and postoperatively; outcomes: speech perception and pure-tone average (PTA) over 3–4 frequencies at time of latest follow-up; timing: from 1946 to March 2021; study design: systematic review.

Articles were imported into the RefWorks citation manager; abstracts were independently evaluated by 2 reviewers. Studies were excluded if they did not include at least one patient with AIED who also received a cochlear implant and had outcomes recorded at a minimum of 3 months following operative date. Review articles and mixed patient population studies which did not allow for AIED-specific patient outcome tracking were excluded. This review was performed in full accordance with PRISMA (Preferred Reporting Items for Systematic Review and Meta-analyses) 2020 guidelines (31), with checklist details in Supplemental Appendix 2, http://links.lww.com/ONO/A0.

### Screening

Full-text articles were evaluated by the same 2 independent reviewers using the exclusion criteria outlined above. Extracted data included number of patients with AIED who underwent CI, adult versus pediatric patient population status, sex, age, and specific AIED etiology (Table 1); data were organized in a spreadsheet using Microsoft Excel, version 16.48. Clinical outcomes evaluated were preoperative and postoperative pure-tone audiometry in quiet and speech perception in quiet at time of latest follow-up (see Supplemental Table 3, http://links.lww.com/ONO/A0). For all included studies, subjective assessment from study authors on respective patient cohort outcomes were also tabulated (see Supplemental Table 3, http://links.lww.com/ONO/A0). Additional data extracted from full text articles included presence of anomalous preoperative radiologic imaging suggesting evidence of AIED activity, incidence of disease activity–related intraoperative technique modification, and postoperative complications (including disease flares, flap complications, surgical site infection, wound healing delay, and need for device removal) (Table 2).

**TABLE 1.** Details for articles (n = 26) included in the present review

First author	Year	Type of study	Country	Patient population	No. AIED patients undergoing CI	Mean age	Sex	AIED etiology	OCEBM grade
Aftab	2010	Retrospective cohort study	USA	Adult	10	49.6	4M/6F	AIED	III
AlHelali	2019	Case report	Saudi Arabia	Adult	1	30	1F	VKH	IV
Bacciu	2015	Case series	Italy	Adult	12	34.1	4M/8F	Cogan syndrome	IV
Bajaj	2012	Retrospective cohort study	UK	Pediatric	3	N/A	N/A	Cogan syndrome	III
Bates	2012	Case report	UK	Pediatric	1	13	1F	CINCA syndrome	IV
Bovo	2012	Case series	Italy	Adult	3	32	3F	Cogan syndrome	IV
Cacco	2020	Case report	Italy	Adult	1	35	1F	EGPA	IV
Cassis	2018	Case report	USA	Adult	1	24	1F	Cogan syndrome	IV
Cheng	2010	Case report	Australia	Adult	1	63	1F	Sweet’s syndrome	IV
Cinamon	1997	Case series	USA	Adult	3	42.3	2M/1F	Cogan syndrome	IV
Cooper	2018	Case series	USA	Adult	11	49.1	N/A	MISSNHL	IV
Forli	2009	Case report	Italy	Adult	1	54	1F	Cogan syndrome	IV
Im	2008	Case report	Korea	Adult	1	25	1F	Cogan syndrome	IV
Kamakura	2017	Case report	USA	Adult	1	64	1M	Cogan syndrome	IV
Kawamura	2010	Case report	Japan	Adult	1	55	1F	Cogan syndrome	IV
Kim	2021	Case series	Korea	Adult	3	19	1M/2F	NLRP3-related AIED	IV
Kontorinis	2010	Retrospective cohort study	Germany	Adult	4	24.4	4F	Cogan syndrome	III
Low	2000	Case report	Singapore	Adult	1	39	1M	Cogan syndrome	IV
Low	2019	Case report	Singapore	Adult	1	23	1F	AIED	IV
Malik	2012	Retrospective cohort study	USA	Adult	26	55	13M/13F	AIED	III
Pasanisi	2003	Prospective cohort study	Italy	Adult	5	36.8	1M/4F	Cogan syndrome	III
Quaranta	2002	Retrospective cohort study	Italy	Adult	5	33.6	3M/2F	AIED	III
Seo	2012	Case report	Korea	Adult	1	34	1M	RP	IV
Sweetow	2005	Case series	USA	Pediatric	1	4	1F	AIED	IV
Vashishth	2018	Retrospective cohort study	Italy	Adult	1	30	1F	Cogan syndrome	III
Wang	2010	Retrospective cohort study	Canada	Adult	25	47.2	7M/18F	AIED	III

AIED indicates autoimmune inner-ear disease; CI, cochlear implantation; CINCA syndrome, chronic infantile neurological cutaneous and articular syndrome; EGPA, eosinophilic granulomatosis with polyangiitis; F, female; M, male; MISSNHL, metachronous idiopathic sudden sensorineural hearing loss; N/A, not available; OCEBM, Oxford Centre for Evidence-Based Medicine; RP, relapsing polychondritis; VKH, Vogt-Koyanagi-Harada.

**TABLE 2.** Perioperative characterization of patients with AIED undergoing CI

	Patients with radiographic findings suggestive of disease activity^a^		Patients with disease activity necessitating intraoperative surgical technique modification^a^					Patients with postoperative complications^a^				
	CT	MRI	Difficult RW Insertion	Partial RW Insertion	Cochlear Drill-out Performed	SV insertion Required	Reoperation Required	Disease Flare	Flap Ischemia	Surgical Site Infection	Wound Healing Delay	Device Removal
Aftab 2010	1	4	—	—	2	—	—	—	—	—	—	—
AlHelali 2019	—	—	—	—	—	1	—	—	—	—	—	—
Bacciu 2015	2	2	—	—	4	2	—	2	—	—	—	—
Bajaj 2012	3	3	—	—	—	—	—	—	—	—	—	—
Bates 2012	—	—	—	—	—	—	—	—	—	—	—	—
Bovo 2012	—	1	—	—	—	1	—	—	—	—	—	—
Cacco 2020	—	—	—	—	—	—	—	—	—	—	—	—
Cassis 2018	—	1	—	—	1	—	—	—	—	—	—	—
Cheng 2010	—	—	—	—	—	—	—	—	—	—	—	—
Cinamon 1997	—	—	—	—	—	—	—	—	—	—	—	—
Cooper 2018	—	—	—	—	—	—	—	—	—	—	—	—
Forli 2009	—	1	1	—	—	—	—	—	—	—	—	—
Im 2008	1	1	—	—	—	—	—	1	—	—	—	—
Kamakura 2017	—	1	1	—	—	—	—	—	—	—	—	—
Kawamura 2010	1	1	—	—	1	—	—	—	—	—	—	—
Kim 2021	—	2	—	—	—	—	—	—	—	—	—	—
Kontorinis 2010	—	—	1	—	—	—	—	1	1	1	1	—
Low 2000	—	—	—	—	1	—	1	—	—	—	1	—
Low 2018	—	—	—	—	—	—	—	1	—	—	—	—
Malik 2012	—	4	—	2	—	—	—	—	—	—	—	—
Pasanisi 2003	1	2	—	—	1	2	—	2	—	—	—	—
Quaranta 2002	1	3	1	—	—	—	—	—	—	—	—	—
Seo 2012	1	—	—	—	—	—	—	—	—	—	—	—
Sweetow 2005	—	1	—	—	—	—	—	—	—	—	—	—
Vashishth 2018	—	—	—	—	1	—	—	—	—	—	—	—
Wang 2010	—	—	—	1	1	—	1	—	1	1	1	2
Total Patients Affected^b^ (n = 124)	11 (8.9)	27 (21.8)	4 (3.2)	3 (2.4)	12 (9.7)	6 (4.8)	2 (1.6)	7 (5.6)	2 (1.6)	2 (1.6)	3 (2.4)	2 (1.6)

a Values reported as No., unless otherwise noted.

b Values reported as No. (%).

— indicates no data available; CT, computer tomography; MRI, magnetic resonance imaging; RW, round window; SV, scala vestibuli.

### Risk of Bias Assessment and Quality Assessment

Two reviewers independently performed a risk of bias assessment using the Joanna Briggs Critical Appraisal Checklist for Case Series and Cohort Studies (32), as seen in Supplemental Appendix 4, http://links.lww.com/ONO/A0. Studies were also evaluated as per the Oxford Centre for Evidence-Based Medicine (OCEBM) grading system (33) in Table 1. All discrepancies were resolved through discussion.

### Meta-analysis

Separate speech perception meta-analyses were conducted for speech recognition scores (SRS) and word recognition scores (WRS). The effect size was measured using the mean difference in scores before and after surgery. The missing standard deviations from some studies were imputed based on similar studies. Effect sizes were calculated as standardized mean differences (SMDs) using Hedge’s *g* as the effect size metric to correct for small-sample bias. To compute the overall mean effect size, we used an inverse-variance random-effects model that assumes the true effects are not identical but normally distributed across studies and allows for random between-study variance in addition to sampling variability (34). To evaluate the percentage of variability of the results attributed to heterogeneity between studies, a statistic for quantifying inconsistency (*I*^2^) was described by expressing the total heterogeneity as a percentage of the total variability and its significance was testing using a χ2 test (35). The following thresholds were used for interpretation of *I*^2^ statistic: 0%–40%, not important; 30%–60%, moderate heterogeneity; 50%–90%, substantial heterogeneity; and 75%–100%, considerable heterogeneity. The funnel plot and Egger’s regression asymmetry test were used to assess publication bias (36). All meta-analyses were performed in R-version-4.0.2 using packages meta (37) and metaphor. Statistical significance was defined as *P* < 0.05.

## RESULTS

### Systematic Review Results

The original search strategy identified 184 studies. Of these, 37 were removed as duplicates and 147 abstracts were screened. Following abstract screening, 46 studies were included, and 26 studies published from 1997 to 2021 ultimately met inclusion criteria. Of the 20 studies excluded during full-text screening, 8 were review articles, 4 did not report CI outcomes, 4 did not report CI outcomes at the 3-month postoperative mark, and 4 were mixed-population studies that did not allow for AIED-specific CI tracking (Fig. 1).

All 26 studies included were full articles (4–12,16–30,38,39). 23.0% (6/26) articles were case series (more than one patient) (10,18,20,24,25,38), 46.2% (12/26) were case reports (one patient) (4,5,9,17,21–23,26–29,39), and 30.8% (8/26) were cohort studies (longitudinal follow-up of one patient group) (6–8,11,12,16,19,30). Of the studies, 73.1% (19/26) studies were based internationally, and 26.9% (7/26) studies were based in the United States (Table 1).

### Quality of Studies

The methodological quality of included studies was modest; studies largely consisted of case reports and case series, with a moderate number of cohort studies (Table 1). Two independent reviewers appraised included studies as having sufficiently low risk of bias for inclusion in this systematic review as per Joanna Briggs criteria (32) (Supplemental Figure 4, http://links.lww.com/ONO/A0). All studies were OCEBM grade III–IV (Table 1). Disparate audiological outcome reporting and follow-up intervals precluded meta-analysis.

### Patient Cohort

Across 26 studies, 124 total patients were included. The mean age at implantation was 26.2 years (range 4–65 years), with 11.5% (3/26) studies focused on the pediatric population and 88.5% (23/26) studies focused on the adult population. Of studies that specified, 58.1% (72/124) patients were female and 30.6% (38/124) were male. Across all studies, 50% (13/26) articles included patients with Cogan’s syndrome, 23.1% (6/26) articles included patients with unspecified causes of AIED, 3.8% (1/26) articles included patients with relapsing polychondritis (RP), 3.8% (1/26) articles included patients with Sweet’s syndrome, 3.8% (1/26) articles included patients with NLRP3-related AIED, 3.8% (1/26) papers included patients with eosinophilic granulomatosis with polyangiitis (EGPA), 3.8% (1/26) papers included patients with Vogt-Koyanagi-Harada (VKH), 3.8% (1/26) articles included patients with metachronous idiopathic sudden SHNL (MISSHNL), and 3.8% (1/26) articles included patients with chronic infantile neurological cutaneous and articular (CINCA) syndrome. The average length of last follow-up after CI was 28.2 months (range 3–120 months). Results were further evaluated for the total cohort of patients (n = 124) (Table 1).

### PTA Outcomes

Of all studies, 84.6% (22/26) reported PTA results for 75% (93/124) patients; 73.1% (19/26) studies reported only preoperative PTA for 71.0% (88/124) patients; 3.8% (1/26) studies reported only postoperative PTA for 2.4% (3/124) patients; and 7.7% (2/26) studies reported both preoperative and postoperative PTA for 1.6% (2/124) patients (see Supplemental Table 3, http://links.lww.com/ONO/A0).

### Speech Perception Outcomes

Twenty-four (92.3%) studies evaluated speech perception for 120 patients. Of all studies, 69.2% (18/26) reported WRS for 62.9% (72/124) patients and 76.9% (20/26) reported sentence recognition scores (SRS) for 93.5% (116/124) patients. Studies either used varied speech perception methods to evaluate patients or did not specify the methods used to evaluate speech perception (see Supplemental Table 3, http://links.lww.com/ONO/A0). Parameters used to assess WRS included: Northwestern University Auditory Test No. 6 (NU-6), free field speech audiometry (FFSA), consonant-nucleus-consonant word list (CNC), phonetically balanced word list (PB), Spondee, Freiburg Monosyllabic word Test (FMT), Arthur Boothroyd word list (AB), and phonetically balanced kindergarten-50 word list (PBK-50). Parameters used to assess SRS included: hearing in noise test (HINT), central institute for the deaf sentence test (CID), Bamford–Kowal–Bench sentence test (BKB), City University of New York sentence test (CUNY), AzBio, Korean central institute for the deaf sentence test (K-CID), and Hochmair–Schulz–Moser sentence test (HSM).

**FIG. 2.**
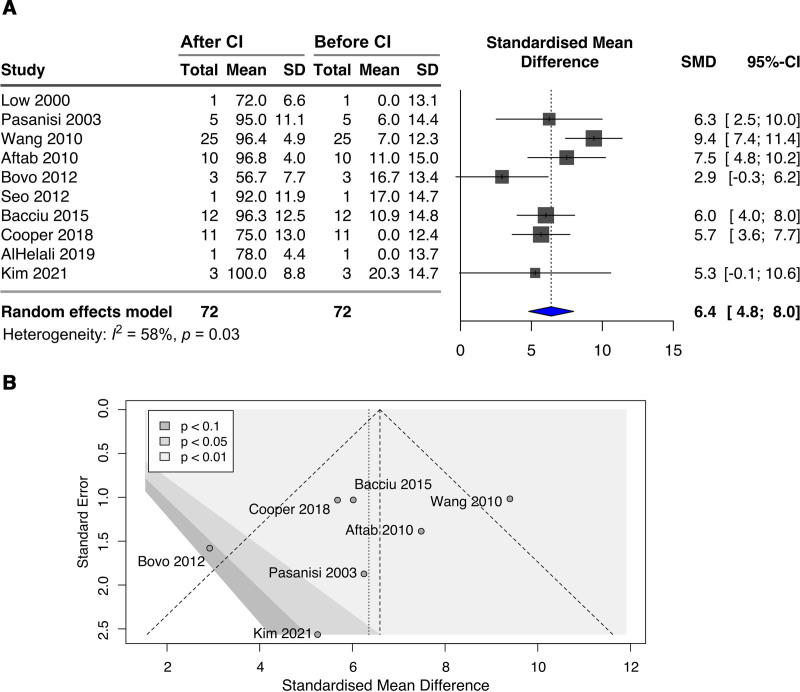
Forest and funnel plots of speech perception speech recognition scores (SRS). *A*, Meta-analysis and forest plots of standardized mean differences (SMD) in sentence recognition scores (SRS) from studies of preoperative and postoperative cochlear implantation speech perception outcomes in patients with AIED. The black boxes indicate the relative sample size; blue diamond: overall standardized mean difference; horizontal lines: 95% confidence intervals. CI indicates cochlear implantation; SD: standard deviation; 95% CI: 95% confidence interval. *B*, Contour-enhanced funnel plots of SMDs in SRS against their standard errors from studies of pre- and post-operative CI. Points represent studies included in the meta-analysis. Shaded contour regions signify the significance level of each study in the plot.

Of the 10 studies (4,7,9,12,16–18,20,25,38) that published preoperative and postoperative SRS speech perception outcomes, seven of them (with more than one case) (7,12,17,18,20,25,38) were pooled for meta-analysis. All patients showed significant improvement in SRS after CI surgery (SMD = 6.4, 95% CI, 4.8-8.0, *P* < 0.0001). There was a substantial heterogeneity in the studies (*I*^2^ = 57.8%, Q [df = 6] = 14.2, *P* = 0.027). The forest plot showing the relative strength of each study included in the meta-analysis is illustrated by Figure 2A. A contour-enhanced funnel plot shows that the data were slightly asymmetrical (Fig. 2B), one small study, Kim et al. (38) lies in the significance region (*P* < 0.05) of the plot, despite having a large standard error. Despite study heterogeneity and data asymmetry, the Egger regression test detected no publication bias in the studies (intercept = –1.89; standard error, 2.12; *P* = 0.41).

Of the 10 studies (4,7,9,10,16,18,21,22,38) that published WRS before and after CI, 4 of them (with more than one case) (7,16,18,38) were combined for meta-analysis. All patients showed significant improvement in WRS after CI surgery (SMD = 5.5, 95% CI, 4.2-6.8, *P* < 0.0001) (40,41). No significant heterogeneity was observed across studies (*I*^2^ = 0%, Q [df = 3] = 0.51, *P* = 0.92), probably due to the low number of studies available; the forest plot showing the relative strength of each study included in the WRS meta-analysis is shown in Supplemental Figure 5, http://links.lww.com/ONO/A0.

### Preoperative Radiologic Manifestations

Of all studies, 57.7% (15/26) reported abnormal preoperative radiographic findings of cochlear disease activity for 30.6% (38/124) patients. Among these, 53.3% (8/15) studies reported abnormal computed tomography (CT) findings for 11 patients, and 93.3 % (14/15) reported abnormal magnetic resonance imaging (MRI) findings for 27 patients (Table 2).

CT findings included ossification of the inferior segment of the cochlear basal turn (7,8,18,19,27,29) and slight cochlear narrowing (9). MRI findings included bilateral enhancement of the cochlear basal turn (8,10,20,22,26,28,38), partial ossification of the membranous labyrinth (27), and cochlear scarring (6).

### Intraoperative Complications

Of all studies, 57.7% (15/26) reported on disease activity necessitating intraoperative surgical technique modification due to degree of cochlear fibrosis and/or ossification for 21.8% (27/124) patients. Among these, 26.7% (4/15) studies reported difficult implant insertion due to round window (RW) pathology for 4 patients; 13.3% (2/15) studies reported partial implant insertion due to RW pathology for 3 patients; 53.3% (8/15) studies reported need for cochlear drill-out due to significant ossification of the inferior basal turn for 12 patients; 26.7% (4/15) studies reported scala vestibuli insertion due to significant round window fibrosis for 6 patients, and 13.3% (2/15) studies reported need for reoperation for 2 patients (Table 2). Reoperation was performed in light of intraoperatively discovered active middle ear disease precluding CI for one patient (4), and device failure 3 years following initial CI for another patient (12).

### Postoperative Complications

In this analysis, 26.9% (7/26) studies reported on postoperative complications from CI with 12.9% (16/124) patients experiencing complications. Among these, 71.4% (5/7) of studies reported on postoperative disease flare occurrences in 5.6% (7/124) of patients; 28.6% (2/7) of studies reported on flap ischemia in 1.6% (2/124) of patients; 28.6% (2/7) of studies reported on surgical site infection for 1.6% (2/124) of patients; 42.9% (3/7) of studies reported on wound healing delay in 2.4% (3/124) of patients; 14.3% (1/7) studies reported on the need for postoperative device removal in 1.6% (2/124) of patients (Table 2).

## DISCUSSION

The present systematic review and narrative synthesis evaluates audiological and surgical management outcomes of AIED patients who underwent CI. To the best of the authors’ knowledge, this is the first systematic review to collate such data.

### Audiological Outcomes

In the present study, we found that patients with AIED demonstrated speech perception improvement following CI. Although no prior studies have evaluated the efficacy of CI in AIED, CI is reported to have relatively high success in AIED patients because this population typically becomes deaf after the age of speech and language skill acquisition, during the “postlingual” period. Another reason why CI is thought to be successful in AIED patients is because these patients are often implanted within 12 months of the onset of profound hearing loss (12,18,29,30,42). Of note, postlingual implantation and early implantation following profound hearing loss are 2 factors that have been highlighted by Blamey et al (43) as being predictive of positive CI outcomes. Postlingually deafened early-implanted AIED patients likely achieve positive CI outcomes because they already have the requisite neurocognitive and neuromuscular skills necessary to hear sound, process language, and express themselves using language (44).

Given that several studies (4,8,20) found decreased audiological outcomes as a consequence of disease activity flares, this suggests that AIED patients may be at risk for long-term degradation in CI function and potentially require long-term outcome monitoring. The pathogenesis of AIED CI outcome degradation is not well understood, but it has been shown that as uncontrolled AIED activity advances, inflammatory processes cause osteogenesis within the cochlea (45); this inflammatory cascade may negatively impact AIED CI outcomes. Interestingly, prior studies have demonstrated that partial cochlear obliteration can occur within 8 weeks of patients’ initial onset of hearing loss (3,46,47). Despite this finding, the best time interval for CI in AIED has not yet been determined. To better contextualize the results of our study, more research is needed to determine how AIED severity and treatment time course is related to CI implant outcomes.

### Preoperative Imaging Outcomes

In this study, a majority of included articles also described the presence of anomalous preoperative radiologic manifestations. We specifically found that 53.8% (14/26) studies reported MRI anomalies, while 30.8% (8/26) studies reported CT anomalies.

Although many studies seem to demonstrate anomalous AIED radiologic manifestations on MRI scans as compared to CT scans, we acknowledge that our imaging data may represent a reporting bias considering all studies did not consistently report findings for both MRI and CT modalities. Nevertheless, it has been suggested that pathologic AIED findings may be better detected with MRI imaging than with CT imaging (8). One study postulates that since AIED leads to degeneration of the inner ear vascular network (48), the resulting reduction in cochlear blood supply promotes formation of metaplastic cochlear bone with deficient mineralization (49). This metaplastic bone may not be detected on CT imaging, potentially increasing the reports of preoperative MRI imaging abnormalities as compared to preoperative CT imaging abnormalities (50).

### Intraoperative Management

Of all studies, 57.7% (15/26) reported the need for disease-driven intraoperative technique adjustment. Typically, CI is carried out through the round window (51). However, patients with AIED often experience ossification of the inferior segment of the basal turn of the cochlea which renders it difficult to perform round window CI (47,52). Although some patients were able to undergo round window insertion despite basal turn ossification, other patients were unable to tolerate complete RW CI. In patients not amenable to complete round window insertion, 3 alternative approaches were pursued. These include incomplete insertion of CI electrodes through the RW, cochlear drill-out to minimize inferior basal turn ossification interference, and CI insertion through the scala vestibuli.

It is important to acknowledge that given the paucity of literature covering CI in AIED, the incidence of round window implantation, partial round window implantation, cochlear drill-out, and scala vestibuli insertion have not been well-characterized in the AIED population. As a result, it is unknown whether the incidence of intraoperative approaches used in AIED CI significantly differs from that of the general population. Although future studies should seek to better characterize the rates of intraoperative technique adjustment in the AIED population, the present study’s results provide preliminary evidence suggesting the need for careful surgical planning in the AIED patient population. Given that AIED processes can complicate the delicate inner ear anatomy and prohibit traditional CI methods, this study emphasizes the importance of considering various intraoperative management options before surgery.

### Postoperative Complications

In the present analysis, 26.9% (7/26) included studies reported the presence of postoperative complications such as disease flares, flap ischemia, surgical site infection, wound healing delay, and need for device removal. AIED flares are hypothesized to result from the surgical stress imposed by CI (7), and occasionally necessitate device removal (12). Furthermore, patients’ long-term immunosuppressive regimens often cause skin atrophy, and are thought to increase the risk of flap ischemia, surgical site infection, and wound healing delay (18). Although the incidence of postoperative complications is not fully characterized in all included articles, to the authors’ knowledge the present study is the first to systematically document postoperative AIED CI complications. Our study highlights the importance of continued research to optimize AIED patient management.

### Limitations

Our results summarize preliminary findings within existing AIED CI literature and highlight the need for consistent reporting of audiological assessment parameters and follow-up intervals (53). Low disease prevalence as well as changes in reporting standards over time have likely contributed to the nonuniformity of AIED CI audiological outcomes.

As seen in other rare disease CI outcome systematic reviews (54), the present review is limited by inconsistent OCEBM-defined methodological quality of included studies (33). Additionally, our analysis is limited by inclusion criteria allowing for study of pediatric and adult AIED CI subjects, which prevents broad generalization of audiological and perioperative management outcomes.

Furthermore, challenges arise at all stages of conducting a meta-analysis on nonrandomized studies on intervention effects, specifically the pre-post design in this study. The SRS and WRS on baseline and post-CI are not independent of each other. Although the value for the correlation should be used in the calculation of the SMD, this value is typically not known. The pre-post design is therefore influenced by natural processes and characteristics of the patients and settings (confounding domain) and suffers from regression to the mean and Hawthorne effects.

### Future Directions

Subsequent studies should aim to explore quality of life (QOL) outcomes in AIED CI using validated assessments such as those developed by McRackan et al (55). Although QOL outcomes following CI have been reported for other inner ear pathology such as Meniere’s disease (54), limited literature exists on the same topic for patients with AIED. QOL measures are important to assess because AIED patients may derive greater benefit from CI beyond just audiological outcome improvement. Tangible QOL improvements could strengthen current management indications for AIED CI and should therefore be investigated.

Another future direction to examine is whether AIED CI speech outcomes remain stable overtime. Existing studies have not systematically characterized whether AIED patients experience long-term speech outcome deterioration or stability. However, given the progressive nature of AIED and reported accounts of CI deterioration (4,8,20), this outcome should be assessed using prospective cohort studies to increase understanding of CI trajectory in AIED.

Despite the present study’s inherent limitations, our review of 124 AIED CI patients provides valuable preliminary insight on audiological and perioperative management outcomes for healthcare professionals who manage AIED. We encourage further exploration of this topic.

## CONCLUSION

In the present systematic review and meta-analysis, we demonstrate that CI in patients with AIED represents a viable treatment for postoperative speech perception outcome improvement. Furthermore, despite a lack of consistent audiological assessment and reporting criteria, a majority of included studies note the presence of anomalous preoperative radiologic manifestations, disease-driven intraoperative technique adjustment, and postoperative complications. Similar to other autoimmune conditions, the management of hearing loss in patients with AIED poses many challenges. Additional research is needed to clarify CI outcomes and perioperative management in this patient population.

## FUNDING SOURCES

None declared.

## CONFLICT OF INTEREST

Michael Hoa, MD, holds the position of Associate Editor for *Otology & Neurotology Open* and has been recused from reviewing or making decisions for the manuscript.

## Data Availability Statement

Data sharing is not applicable to the article as no datasets were generated. Publicly available studies were analyzed in this report (4–12,16–30).

## Supplementary Material


