# Supplementary material for: Cochlear Implantation and Perioperative Management in Autoimmune Inner Ear Disease: A Systematic Review and Meta-Analysis
Source: Otol Neurotol Open. 2021 Dec 9;1(2):e006. doi: 10.1097/ONO.0000000000000006 (PMC10969513; doi:10.1097/ONO.0000000000000006)

Appendix

Search strategies for PubMed, Medline Ovid, Ovid Embase, and CINAHL:

PubMed:

(“Autoinflammatory disorder”[Mesh] OR “autoimmune disease”[Mesh] OR “Cogan’s syndrome”[Mesh] OR “cogan syndrome” OR “Sweet’s disease” OR “Sweet syndrome” OR “relapsing polychondritis”[Mesh] OR “inflammatory bowel disease” [Mesh]) AND (“cochlear implant”[Mesh] OR “cochlear implants”[Mesh] OR “cochlear implantation”[Mesh] OR “cochlear implantations”[Mesh] OR “correction of hearing impairment”[Mesh] OR “cochlear prosthesis”[Mesh] OR “auditory prosthesis”[Mesh])

Medline Ovid:

1. AIED.mp
2. autoimmune inner ear disease.mp
3. auto-immune inner ear disease.mp
4. IMIED.mp
5. immune-mediated inner ear disease.mp
6. autoinflammatory disorder.mp
7. Autoinflammatory disorder.mp
8. autoimmune disease.mp
9. Cogan’s syndrome.mp
10. cogan syndrome.mp
11. Sweet’s disease.mp
12. Sweet syndrome.mp
13. relapsing polychondritis.mp
14. inflammatory bowel disease.mp
15. extraintestinal crohn’s.mp
16. 1 or 2 or 3 or 4 or 5 or 6 or 7 or 8 or 9 or 10 or 11 or 12 or 13 or 14 or 15
17. cochlear implant.mp
18. cochlear implants.mp
19. cochlear implantation.mp
20. cochlear implantations.mp
21. correction of hearing impairment.mp
22. cochlear prosthesis.mp
23. auditory prosthesis.mp
24. 17 or 18 or 19 or 20 or 21 or 22 or 23
25. 16 and 24

Ovid Embase:

1. AIED.mp
2. autoimmune inner ear disease.mp
3. auto-immune inner ear disease.mp

4. IMIED.mp
5. immune-mediated inner ear disease.mp
6. autoinflammatory disorder.mp
7. Autoinflammatory disorder.mp
8. autoimmune disease.mp
9. Cogan's syndrome.mp
10. cogan syndrome.mp
11. Sweet's disease.mp
12. Sweet syndrome.mp
13. relapsing polychondritis.mp
14. inflammatory bowel disease.mp
15. extraintestinal crohn's.mp
16. 1 or 2 or 3 or 4 or 5 or 6 or 7 or 8 or 9 or 10 or 11 or 12 or 13 or 14 or 15
17. cochlear implant.mp
18. cochlear implants.mp
19. cochlear implantation.mp
20. cochlear implantations.mp
21. correction of hearing impairment.mp
22. cochlear prosthesis.mp
23. auditory prosthesis.mp
24. 17 or 18 or 19 or 20 or 21 or 22 or 23
25. 16 and 24

CINAHL:

1. AIED.mp
2. autoimmune inner ear disease.mp
3. auto-immune inner ear disease.mp
4. IMIED.mp
5. immune-mediated inner ear disease.mp
6. autoinflammatory disorder.mp
7. Autoinflammatory disorder.mp
8. autoimmune disease.mp
9. Cogan's syndrome.mp
10. cogan syndrome.mp
11. Sweet's disease.mp
12. Sweet syndrome.mp
13. relapsing polychondritis.mp
14. inflammatory bowel disease.mp
15. extraintestinal crohn's.mp
16. 1 or 2 or 3 or 4 or 5 or 6 or 7 or 8 or 9 or 10 or 11 or 12 or 13 or 14 or 15
17. cochlear implant.mp
18. cochlear implants.mp
19. cochlear implantation.mp
20. cochlear implantations.mp
21. correction of hearing impairment.mp

22. cochlear prosthesis.mp
23. auditory prosthesis.mp
24. 17 or 18 or 19 or 20 or 21 or 22 or 23
25. 16 and 24

PRISMA 2020 Checklist

Section and Topic	Item #	Checklist item	Page # or Location where item is reported
**TITLE**			
Title	1	Identify the report as a systematic review.	Title Page
**ABSTRACT**			
Abstract	2	See the PRISMA 2020 for Abstracts checklist.	1
**INTRODUCTION**			
Rationale	3	Describe the rationale for the review in the context of existing knowledge.	2
Objectives	4	Provide an explicit statement of the objective(s) or question(s) the review addresses.	2
**METHODS**			
Eligibility criteria	5	Specify the inclusion and exclusion criteria for the review and how studies were grouped for the syntheses.	3
Information sources	6	Specify all databases, registers, websites, organisations, reference lists and other sources searched or consulted to identify studies. Specify the date when each source was last searched or consulted.	3
Search strategy	7	Present the full search strategies for all databases, registers and websites, including any filters and limits used.	Appendix 1
Selection process	8	Specify the methods used to decide whether a study met the inclusion criteria of the review, including how many reviewers screened each record and each report retrieved, whether they worked independently, and if applicable, details of automation tools used in the process.	3
Data collection process	9	Specify the methods used to collect data from reports, including how many reviewers collected data from each report, whether they worked independently, any processes for obtaining or confirming data from study investigators, and if applicable, details of automation tools used in the process.	4
Data items	10a	List and define all outcomes for which data were sought. Specify whether all results that were compatible with each outcome domain in each study were sought (e.g. for all measures, time points, analyses), and if not, the methods used to decide which results to collect.	4, Table 2
	10b	List and define all other variables for which data were sought (e.g. participant and intervention characteristics, funding sources). Describe any assumptions made about any missing or unclear information.	4, Table 2
Study risk of bias assessment	11	Specify the methods used to assess risk of bias in the included studies, including details of the tool(s) used, how many reviewers assessed each study and whether they worked independently, and if applicable, details of automation tools used in the process.	4, Supplemental Digital Content 4
Effect measures	12	Specify for each outcome the effect measure(s) (e.g. risk ratio, mean difference) used in the synthesis or presentation of results.	5-6
Synthesis methods	13a	Describe the processes used to decide which studies were eligible for each synthesis (e.g. tabulating the study intervention characteristics and comparing against the planned groups for each synthesis (item #5)).	5-6
	13b	Describe any methods required to prepare the data for presentation or synthesis, such as handling of missing summary statistics, or data conversions.	5-6
	13c	Describe any methods used to tabulate or visually display results of individual studies and syntheses.	5-6
	13d	Describe any methods used to synthesize results and provide a rationale for the choice(s). If meta-analysis was performed, describe the model(s), method(s) to identify the presence and extent of statistical heterogeneity, and software package(s) used.	5-6
	13e	Describe any methods used to explore possible causes of heterogeneity among study results (e.g. subgroup analysis, meta-regression).	5-6
	13f	Describe any sensitivity analyses conducted to assess robustness of the synthesized results.	5-6
Reporting bias	14	Describe any methods used to assess risk of bias due to missing results in a synthesis (arising from reporting biases).	4,

PRISMA 2020 Checklist

Section and Topic	Item #	Checklist item	Page # or Location where item is reported
assessment			Supplemental Digital Content 4
Certainty assessment	15	Describe any methods used to assess certainty (or confidence) in the body of evidence for an outcome.	5-6
**RESULTS**			
Study selection	16a	Describe the results of the search and selection process, from the number of records identified in the search to the number of studies included in the review, ideally using a flow diagram.	Figure 1
	16b	Cite studies that might appear to meet the inclusion criteria, but which were excluded, and explain why they were excluded.	N/A
Study characteristics	17	Cite each included study and present its characteristics.	Table 1
Risk of bias in studies	18	Present assessments of risk of bias for each included study.	Supplemental Digital Content 4
Results of individual studies	19	For all outcomes, present, for each study: (a) summary statistics for each group (where appropriate) and (b) an effect estimate and its precision (e.g. confidence/credible interval), ideally using structured tables or plots.	Table 2
Results of syntheses	20a	For each synthesis, briefly summarise the characteristics and risk of bias among contributing studies.	Supplemental Digital Content 4
	20b	Present results of all statistical syntheses conducted. If meta-analysis was done, present for each the summary estimate and its precision (e.g. confidence/credible interval) and measures of statistical heterogeneity. If comparing groups, describe the direction of the effect.	8-9
	20c	Present results of all investigations of possible causes of heterogeneity among study results.	8-9
	20d	Present results of all sensitivity analyses conducted to assess the robustness of the synthesized results.	8-9
Reporting biases	21	Present assessments of risk of bias due to missing results (arising from reporting biases) for each synthesis assessed.	8-9
Certainty of evidence	22	Present assessments of certainty (or confidence) in the body of evidence for each outcome assessed.	8-9
**DISCUSSION**			
Discussion	23a	Provide a general interpretation of the results in the context of other evidence.	9-14
	23b	Discuss any limitations of the evidence included in the review.	13-14
	23c	Discuss any limitations of the review processes used.	14
	23d	Discuss implications of the results for practice, policy, and future research.	14-15
**OTHER INFORMATION**			
Registration and protocol	24a	Provide registration information for the review, including register name and registration number, or state that the review was not registered.	3
	24b	Indicate where the review protocol can be accessed, or state that a protocol was not prepared.	3
	24c	Describe and explain any amendments to information provided at registration or in the protocol.	N/A
Support	25	Describe sources of financial or non-financial support for the review, and the role of the funders or sponsors in the review.	N/A

PRISMA 2020 Checklist

Section and Topic	Item #	Checklist item	Page # or Location where item is reported
Competing interests	26	Declare any competing interests of review authors.	N/A
Availability of data, code and other materials	27	Report which of the following are publicly available and where they can be found: template data collection forms; data extracted from included studies; data used for all analyses; analytic code; any other materials used in the review.	N/A

From: Page MJ, McKenzie JE, Bossuyt PM, Boutron I, Hoffmann TC, Mulrow CD, et al. The PRISMA 2020 statement: an updated guideline for reporting systematic reviews. BMJ 2021;372:n71. doi: 10.1136/bmj.n71

For more information, visit: http://www.prisma-statement.org/

TABLE. Audiometric and speech perception outcomes before and after CI in patients with AIED.

	Patients (n)	Mean Pure Tone Average			Mean Speech Perception					
		Before CI	After CI	Last Follow-Up Interval Post-CI	Test	Assessment Parameter	Before CI[c]	After CI[c]	Last Follow-Up Interval Post-CI	Qualitative Outcome of CI in AIED
**Aftab 2010**	10	102 ± 18 dB HL	-	-	WRS	NU-6	11 ± 17	87.2 ± 11	>12 months	CI is an "…effective option for hearing restoration in patients with AIED…", however, it should be considered at an earlier timepoint due to risk of cochlear fibrosis or ossification.
					SRS	HINT, CID[a]	11 ± 15	96.8 ± 4		
**AlHelali 2019**	1	107 dB HL	-	-	SRS	N/S	0	78	5 years	"[CI] is useful to improve hearing in VKH patients."
**Bacciu 2015**	12	> 90 dB HL	-	-	WRS	N/S	9.7	94	5 years	"…patients with CS receive significant… benefits from…" CI.
					SRS	N/S	10.9	96.3		
**Bajaj 2012**	3	-	35-40 dB HL	10 years	-	-	-	-	10 years	"Good audiological and speech out-comes…" following CI.
**Bates 2012**	1	90 dB HL	-	-	SRS	BKB	-	100	3 years	Patient able to "…attend mainstream school…" with "…improved academic performance."
**Bovo 2012**	3	Profound bilateral deafness	-	-	SRS	N/S	16.7	56.7	3.5 years	Overall benefit, however, two patients had delayed non-satisfactory outcomes due to disease flares.
**Cacco 2020**	1	100 dB HL	30 dB HL	2 years	WRS	FFSA	0	50	2 years	CI represents a "…viable treatment in stabilized…" EGPA-related AIED.
**Cassis 2018**	1	Profound bilateral deafness	-	-	WRS	N/S	0	76	5 months	The patient "…consistently improved with [CI] overtime."
**Cheng 2010**	1	90 dB HL	-	-	SRS	CUNY	-	99	3 months	"CI can successfully restore hearing when immunotherapy fails…" in Sweet's disease.
**Cinamon 1997**	3		-	-	WRS	N/S	-	81.7	2 years	

		Profound bilateral deafness			SRS	N/S	-	91	2 years	Patients "…gained a great benefit from [CI]."
**Cooper 2018**	11	87 dB HL	-	1 year	SRS	AzBio	0	91 ± 12	1 year	"CI can successfully restore hearing in individuals who do not experience [medical treatment] recovery."
					SRS	HINT	0	75 ± 13	1 year	
**Forli 2009**	1	90 dB HL	-	-	WRS	N/S	-	98	3 years	Patients "…achieved excellent post implant results."
					SRS	N/S	-	98	3 years	
**Im 2008**	1	-	-	-	WRS	N/S	-	91	1 year	"CI is an excellent treatment modality for the profound hearing loss found in Cogan's syndrome."
					SRS	N/S	-	96	1 year	
**Kamakura 2017**	1	90 dB HL	-	-	WRS	CNC	-	56	1 year	"Postoperative hearing [outcomes are] reported to be good to excellent."
**Kawamura 2010**	1	115 dB HL	-	-	WRS	N/S	-	78	1 year	"[CI] dramatically improves speech perception in patients with bilateral profound SNHL due to CS."
					SRS	N/S	-	79	1 year	
**Kim 2021**	3	-	-	-	WRS	PB (kids)	15	92.5	1.3 years	"[CI] is a viable treatment option [for AIED]."
					WRS	Spondee (adults)	25	95		
					SRS	K-CID	20.3	100		
**Kontorinis 2010**	4	92 dB HL	-	-	WRS	FMT	-	70	9.25 years	"CI is the appropriate hearing rehabilitation method in Cogan syndrome patients."
					SRS	HSM	-	99.1		
**Low 2000**	1	-	-	-	WRS	AB	0	31	3 months	"Successful CI [is described] in a patient with Cogan's syndrome complicated by chronic ear disease."
					SRS	BKB	0	72		
**Low 2019**	1	100 dB HL	-	-	-	-	-	-	3 years	"Patient experienced a deterioration of an initially good post-implant outcome."
**Malik 2012**	26	-	-	-	WRS	CNC	-	51	17 months	"Early intervention with CI…offers the opportunity to restore hearing function and communication capacity…".
					SRS	HINT	-	82		

**Pasanisi 2003**	5	Profound bilateral deafness	-	-	WRS	N/S	3.4	91.4	1 year	"Our five patients achieved high levels of speech understanding after undergoing CI…".
					SRS	N/S	6	95		
**Quaranta 2002**	5	100 dB HL	-	-	WRS	N/S	-	79	2 years	"CI in patients affected by immune-mediated inner ear disorders is effective…".
					SRS	N/S	-	89		
**Seo 2012**	1	110 dB HL	40 dB HL	3 months	WRS	N/S	0	90	4 months	"CI [remains] a good optional treatment for IMIED, including RP…".
					SRS	N/S	17	92		
**Sweetow 2005**	1	Profound bilateral deafness	-	-	WRS	PBK-50	0	82	7 months	"Improved auditory skills were primarily from … [CI]."
**Vashishth 2018**	1	Profound bilateral deafness	-	-	WRS	N/S	-	52	4 years	"CI in cochlear ossification is feasible…".
					SRS	N/S	-	62		
**Wang 2010**	25	>70 dB HL	-	-	SRS	CID, CUNY, HINT[b]	7.0 ± 12.3	96.4 ± 4.9	>2 years	"CI provides a therapeutic option for patients with autoimmune inner ear disease who would otherwise have a poor prognosis for hearing improvement."

CI indicates cochlear implantation; AIED, autoimmune inner-ear disease; N/S, not specified; WRS, word recognition score; SRS, sentence recognition score; SD, standard deviation; NU-6, Northwestern University Auditory Test Number 6; HINT, hearing in noise test; CID, central institute for the deaf sentence test; dB, decibels; HL, hearing loss; -, no data available; VKH, Vogt-Koyanagi-Harada disease; CS, Cogan syndrome; BKB, Bamford-Kowal-Bench sentence test; FFSA, free field speech audiometry; CUNY, City University of New York sentence test; CNC, consonant-nucleus-consonant word list; CS, cogan syndrome; SNHL, sensorineural hearing loss; PB, phonetically balanced word list; K-CID, Korean version-central institute for the deaf sentence test; FMT, Freiburg Monosyllabic word Test; HSM, Hochmair-Schulz-Moser sentence test; AB, Arthur Boothroyd word list; AID, autoimmune disease; SNHL, sensorineural hearing loss; RP, relapsing polychondritis; PBK-50, phonetically balanced kindergarten-50 word list.

[a]HINT for all patients, except for 3 patients for whom CID was used; individual test scores were not reported

[b]CID, CUNY, and HINT results were collectively reported in this study; individual test scores were not reported

[c]Values are reported as mean, or mean ± SD when SD data available in original paper

Appendix

Risk of bias for 26 included studies using Joanna Briggs Critical Appraisal Checklist. Studies were evaluated by two independent reviewers. Green: yes; red: no; yellow: unclear; grey: not applicable.

Yes / No / Maybe / Not Applicable	Were there clear criteria for inclusion in the study?	Was the condition measured in a standard, reliable way for all included participants?	Were valid methods used for identification of the condition for each participant?	Did the study have consecutive inclusion of participants?	Did the study have complete inclusion of participants?	Was there clear reporting of the demographics of the participants in this study?	Was there clear reporting of clinical information of the participants?	Were the outcomes for follow-up results of cases clearly reported?	Was there clear reporting of the presenting site(s)/clinic(s) demographic information?	Was statistical analysis appropriate?
Aftab 2010	Yes	Yes	Yes	Yes	Yes	Yes	Yes	Yes	Yes	Yes
AlHelali 2019	Yes	Not Applicable	Yes	Not Applicable	Not Applicable	Yes	Yes	Maybe	Maybe	Not Applicable
Bacciu 2015	Yes	Yes	Yes	Yes	Yes	Yes	Yes	Yes	Yes	Yes
Bajaj 2012	Yes	Yes	Maybe	Yes	Yes	Yes	Yes	Yes	Yes	Yes
Bates 2012	Yes	Not Applicable	Yes	Not Applicable	Not Applicable	Yes	Yes	Yes	Yes	Not Applicable
Bovo 2012	Yes	Yes	Yes	Yes	Yes	Yes	Yes	Yes	Yes	Yes
Cacco 2020	Yes	Not Applicable	No	Not Applicable	Not Applicable	Yes	Yes	Maybe	Yes	Not Applicable
Cassis 2018	Yes	Not Applicable	Yes	Not Applicable	Not Applicable	Yes	Yes	Yes	Yes	Not Applicable
Cheng 2010	Yes	Not Applicable	Yes	Not Applicable	Not Applicable	Yes	Yes	Yes	Yes	Not Applicable
Cinamon 1997	Yes	Not Applicable	Yes	Not Applicable	Not Applicable	Yes	Yes	Yes	Yes	Not Applicable
Cooper 2018	Yes	Yes	Yes	Yes	Yes	Yes	Yes	Yes	Yes	Yes
Forli 2009	Yes	Not Applicable	Yes	Not Applicable	Not Applicable	Yes	Yes	Yes	Yes	Not Applicable
Im 2008	Yes	Not Applicable	Maybe	Not Applicable	Not Applicable	Yes	Yes	Yes	Yes	Not Applicable
Kamakura 2017	Yes	Not Applicable	Yes	Not Applicable	Not Applicable	Yes	Yes	Yes	Maybe	Not Applicable
Kawamura 2010	Yes	Not Applicable	Yes	Not Applicable	Not Applicable	Yes	Yes	Yes	Yes	Not Applicable
Kim 2021	Yes	Not Applicable	Yes	Not Applicable	Not Applicable	Yes	Yes	Yes	Yes	Not Applicable
Kontorinis 2010	Yes	Yes	Yes	Yes	Yes	Yes	Yes	Yes	Yes	Yes
Low 2000	Yes	Not Applicable	Yes	Not Applicable	Not Applicable	Yes	Yes	Yes	Maybe	Not Applicable
Low 2019	Yes	Not Applicable	Yes	Not Applicable	Not Applicable	Yes	Yes	Maybe	Yes	Not Applicable
Malik 2012	Yes	Yes	Yes	Yes	Yes	Yes	Yes	Yes	Yes	Yes
Pasanisi 2003	Yes	Yes	Yes	Yes	Yes	Yes	Yes	Yes	Yes	Yes
Quaranta 2002	Yes	Yes	Yes	No	Maybe	Yes	Yes	Yes	Yes	Yes
Seo 2012	Yes	Not Applicable	Yes	Not Applicable	Not Applicable	Yes	Yes	Yes	Yes	Not Applicable
Sweetow 2005	Yes	Not Applicable	Yes	Not Applicable	Not Applicable	Yes	Yes	Yes	Yes	Not Applicable
Vashishth 2018	Yes	Yes	Yes	No	No	Yes	Yes	Yes	Maybe	Yes
Wang 2010	Yes	Yes	Yes	Yes	Yes	Yes	Yes	Yes	Yes	Yes

Study	After CI Total	After CI Mean	After CI SD	Before CI Total	Before CI Mean	Before CI SD	SMD	95%-CI
Low 2000	1	31.0	13.9	1	0.0	21.9		
Pasanisi 2003	5	91.4	9.8	5	3.4	14.9	6.3	[2.5; 10.1]
Sweetow 2005	1	82.0	10.5	1	0.0	16.2		
Aftab 2010	10	87.2	11.0	10	11.0	17.0	5.1	[3.1; 7.1]
Seo 2012	1	90.0	10.8	1	0.0	16.7		
Bacciu 2015	12	94.0	10.8	12	9.7	16.6	5.8	[3.8; 7.8]
Cassis 2018	1	76.0	11.5	1	0.0	17.8		
Cacco 2020	1	50.0	12.2	1	0.0	19.1		
Kim 2021, PB	2	92.5	11.4	2	15.0	17.7	3.0	[-14.0; 19.9]
Kim 2021, Spondee	1	95.0	11.6	1	25.0	18.0		
**Random effects model**	**35**			**35**			**5.5**	**[4.2; 6.8]**

Heterogeneity: $I^2 = 0\%$, $p = 0.92$

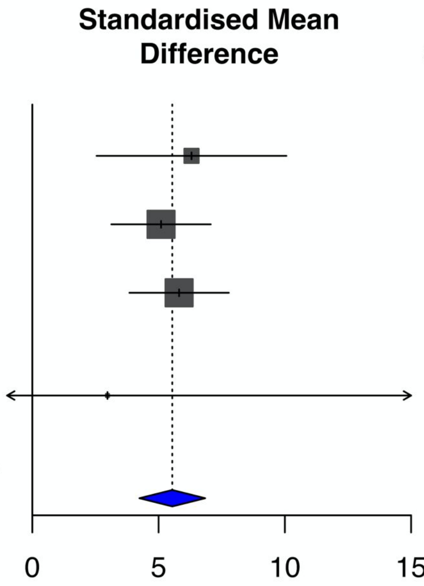

Supplement: Supplementary file 1 [file ono-1-e006-s001.pdf]
